# The Uncontrolled Manifold Concept Reveals That the Structure of Postural Control in Recurve Archery Shooting Is Related to Accuracy

**DOI:** 10.3390/jfmk3030048

**Published:** 2018-09-17

**Authors:** Ben Serrien, Elout Witterzeel, Jean-Pierre Baeyens

**Affiliations:** 1 Vrije Universiteit Brussel, Faculteit Lichamelijke Opvoeding en Kinesitherapie, dept. Movement and Sport Sciences, 1050 Brussels, Belgium; 2 Universiteit Antwerpen, Faculteit Toegepaste Ingenieurswetenschappen, dept. Electronics and ICT, 2000 Antwerp, Belgium; 3 Thim Van Der Laan University College Physiotherapy, 7302 Landquart, Switzerland

**Keywords:** archery, shooting, postural control, accuracy, uncontrolled manifold, motor abundance

## Abstract

In this study, we examine the structure of postural variability in six elite-level recurve archers using the uncontrolled manifold concept. Previous research showed equivocal results for the relationship between postural control and shooting accuracy, but these studies were mainly limited to a descriptive approach to postural variability/stability and did not take the simultaneous movements of the upper limb joints into account. In this study, we show that the goal-equivalent variability which stabilizes the orientation of the arrow in space is significantly larger than that of the non-goal-equivalent variability in arrows of high accuracy (score 9 or 10). Conversely, arrows of lower accuracy (score 6, 7, or 8) failed to reach significant thresholds throughout the majority of the aiming phase. This analysis reveals that it is not necessary (or even possible) for elite archers to minimize the movements of all degrees of freedom during aiming, but rather that the structure of variability of the redundant kinematic chain is exploited so that the relevant performance variable (orientation of the arrow) is stabilized.

## 1. Introduction

Recurve archery is an Olympic sport where the athlete has to aim the arrow very accurately to the target. This process requires quasi-static control of posture and the upper limbs while withstanding considerable forces from the bow and string. Previous research in archery examined and identified multiple accuracy-related movement characteristics like muscle activity patterns of the forearm [1,2,3,4], releasing the arrow within the ST-phase of the cardiac cycle [5], timing of several key actions and phases [6], special skills related to specificity of the target distance [7], and postural control.

With regards to the latter, previous research in postural control of archery yielded different results about its relationship with shooting accuracy. A study with Malaysian archers revealed that the magnitude of postural sway during the set-up phase was negatively correlated with performance (r = −0.22), while sway during the release phase was positively correlated with performance (r = 0.25) and sway during the aiming phase was uncorrelated (r = −0.02) [8]. A multilinear regression analysis showed that a reduction in maximum sway speed post release combined with a reduced clicker reaction time and increased bow draw force had moderate predictive power for shooting accuracy (*R*^2^ = 0.42) for international elite-level archers [9]. Tinazci reported that postural sway amplitude and velocity were positively correlated with accuracy (0.14 < r < 0.22), while aiming sway was negatively correlated (−0.11 < r < −0.13) [10]. Stuart and Atha [11] observed that inter-trial consistency in positions of markers on the head, elbow, and bow could not be used to distinguish shooting accuracy for elite-level archers.

A classical reasoning seen in many studies on postural control in archery and other precision shooting sports is that the postural sway and limb movements should be minimized. In first approximation, this is of course correct, but it should be acknowledged that they can never reach a globally stable minimum of zero movement due to cardiac and breathing cycles and small unpredictable fluctuations in the external force on the bow and string hands due to a non-constant hand position and muscle tremors. However, these studies usually approach postural control using measures like center-of-pressure or center-of-mass sway and velocities [8,9,10] without simultaneous analysis of the bow- and string-arm joint kinematics. Other approaches include the assessment of stability of selected points on the body [11]; however, here, the same problem arises that they are not assessed simultaneously in their ability to stabilize the orientation of the arrow. The real variable to be minimized is the sway of the orientation of the arrow with respect to the target which depends, in a non-linear way, on the simultaneous states of posture (lower limb stance and pelvis and trunk orientations) and orientation of the three major joints of the upper limb.

**Figure 1:**
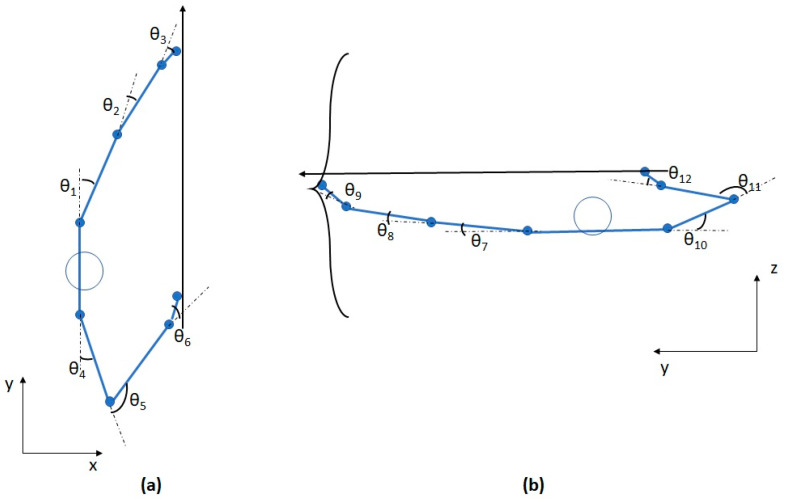
Marker placement and definitions of joint angles in the horizontal plane (**a**) and vertical plane (**b**). Positions are exaggerated to illustrate the definition of the joint angles. The positions shown here represent positive joint angles. The combined orientation of the 12 joint angles and positions of the acromion markers (which reflect the postural orientation of the lower limbs and the trunk) define the approximate orientation of the arrow in space (the exact orientation also depends on the bow geometry and where they hold the bow, but we assume the approximate and exact orientations to be fixed with respect to each other).

The uncontrolled manifold (UCM) concept is a tool that allows the simultaneous assessment of motor control of many degrees of freedom in their cooperation to stabilize a hypothetical performance variable [12]. The UCM methodology is tightly linked to the motor synergy concept [13,14,15]. Synergy can be defined at several levels of the movement system (e.g., kinematic, muscular, or neural levels), and is always associated with multiple elements (the degrees of freedom at every level) that work together to achieve a certain common goal [16]. Synergies, thus, represent a systematic covariation between degrees of freedom to stabilize a task-specific performance variable. Movement in one degree of freedom can be counterbalanced by covariance from movement in other degrees of freedom, resulting in kinematic synergy. The UCM methodology can quantify the strength of synergy with respect to hypothetical performance variables. For the present application to archery shooting, the most relevant performance variable for accuracy is the orientation of the arrow in space, which can be accomplished by multiple segment and joint configurations. For instance, a small reduction in right-shoulder horizontal abduction (θ_4_, see Figure 1) can lead to a net zero change in right-hand position by simultaneous appropriate covariations in the right elbow (θ_5_) and wrist angles (θ_6_).

The UCM approach was previously successful in explaining motor control in various movements (for a review, see References [13,17]), including pistol shooting [18]. The latter study analyzed how the movements of the seven degrees of freedom of the arm were structured in order to control the orientation of the pistol barrel with respect to the target. The several degrees of freedom showed systematic covariations that kept this performance variable stable from trial to trial, while the movement patterns of the individual degrees of freedom varied. These results are not directly transferable to archery because the task in this study was a quick-draw shoot-out, and thus, without a real stable aiming phase. However, the approach with the UCM is attractive for the problem of postural control in archery shooting because the orientation of the arrow depends on the simultaneous actions of several degrees of freedom of both the arms and trunk in a non-linear way.

Therefore, in the present study, we aimed to provide a deeper insight into the relationship between postural control and shooting accuracy by testing specific hypotheses within the uncontrolled manifold concept [12]. This method allows taking specific covariations between the degrees of freedom of a redundant kinematic chain into account in the assessment of achieving success in selected performance variables.

## 2. Materials and Methods

### 2.1. Subjects

Six elite archers from a national team participated in this study (five males, one female, ages 16–45). Each participant was at least one year active as an elite archer in international competitions, and all were right-handed (drawing arm). This study was approved by the Brussels University Hospital Ethical Commission and informed consent was signed by all participants.

### 2.2. Task and Measurements

All archers completed 100 shots to an 18-m indoor target with their own bows. The target was a standard FITA (World Archery federation) circle of 40-cm diameter, located 130 cm above the ground. The archers were instructed to try to hit the center of the target on each shot. Accuracy of the shot was marked according to the standard rules as 10, 9, 8, 7, 6, or 0, starting from the center (10) to the outer ring (6) or miss (0). The first 10 shots were used to accommodate the archers to the room and to the measurement equipment, and the final 90 shots were used for data analysis. Archers shot 10 rounds of 10 arrows with a 3-min rest between rounds to prevent potential effects of fatigue. They were equipped with eight retroreflective markers (14 mm) on the (bilateral) acromia, epicondylus lateralis humeri, processus styloidueus radii, and caput metacarpale II, which were captured at 50 Hz with a 5-camera VICON MX F-20 system. Marker trajectories were labeled and filtered (low-pass Butterworth filter, fourth-order zero-lag, 6-Hz cut-off frequency) in the VICON Nexus 1.8.2 software. The global reference frame had its origin at the line of shooting and was oriented with the *y*-axis toward the target, the *x*-axis to the right, and the *z*-axis upward.

### 2.3. Data Pre-Processing

Marker trajectories were imported into MATLAB R2015a for kinematic analysis. Joint angles were defined for the shoulder, elbow, and wrist in the horizontal and vertical planes, as shown in Figure 1. The joint angles were calculated with the standard cosine rule between the vectors forming the longitudinal orientations of the segments. The analysis in the two-dimensional (2D) planes was a simplified version of the full three-dimensional (3D) model, but makes the mathematical formulation more tractable and allows distinguishing postural control of aiming in different directions. Previous research showed that postural control during aiming tasks in antero-posterior and mediolateral directions is controlled by different processes [19], which we also wanted to take into account in the present model.

**Figure 2:**
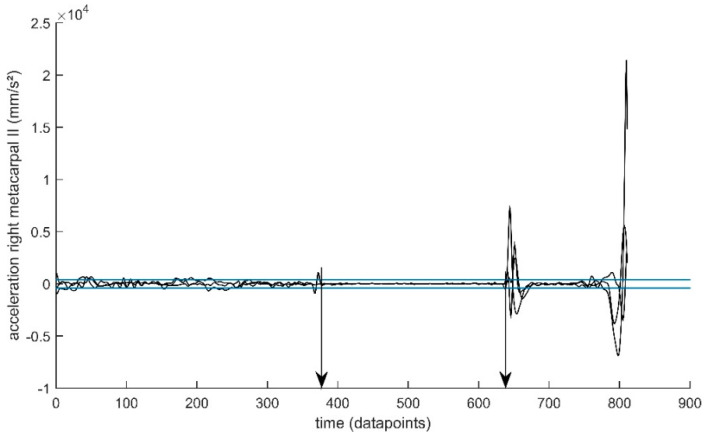
Time normalization of the aiming phase. When all components of the acceleration of the right-hand metacarpal II (black traces) remained between −400 and 400 mm/s^2^ (blue lines) for at least five consecutive time samples, the start of the aiming phase was defined (left vertical arrow), and release was defined when this condition was no longer met (right vertical arrow).

Because the VICON system could not be synchronized with the clicker from the bows, we defined the aiming phase of the archers from the kinematic data. We used the acceleration of the right-hand metacarpal II to define onset of the aiming phase and arrow release, using a 400-mm/s^2^ threshold and a 0.1-s time window (see Figure 2). The detection of onset and release was verified visually for each shot and compared to the VICON images. In case the detection showed an obvious error, a lower or higher threshold was used or the time window was changed, but this was only necessary in a few trials. All kinematic variables were time-normalized to 101 data points to analyze the structure of the variability throughout the aiming phase (0–100%).

### 2.4. Uncontrolled Manifold (UCM) Analysis

The UCM concept [12] assumes that the variability in a system with abundant degrees of freedom is structured in a specific way to stabilize a particular performance variable. In the present study of archery shooting, the accuracy of the shot depends critically on the relative orientation of both hands which will, therefore, be used as hypothetical performance variable. This variable was previously successful in explaining motor control in a pistol shooting task [18]. The geometric model of Figure 1 shows the dependency of this performance variable on several elemental variables, namely the orientation of the trunk (described by the position of the acromion markers) and the shoulder, elbow, and wrist angles. For the UCM analysis in this study, we hypothesized that the performance variable was the relative orientation between both end-effectors (metacarpal markers), as a proxy for the true orientation of the arrow. The end-effector positions of the left (*LH*) and right (*RH*) hand were written as geometric models in the horizontal plane (Equations (1) and (2)) and in the vertical plane (Equations (3) and (4)) using the joint angles (*θ_i_*) and the acromion positions (*RACR*, *LACR*) as the elemental variables (*l_i_* represents the segment lengths of the upper arm (1), lower arm (2), and hand (3).

(1) $$\begin{array}{c} \left(\begin{array}{c} LH_{x}\\ LH_{y} \end{array}\right)=\left(\begin{array}{c} LACR_{x}+l_{1}sin\left(\theta_{1}\right)+l_{2}sin\left(\theta_{1}+\theta_{2}\right)+l_{3}sin\left(\theta_{1}+\theta_{2}+\theta_{3}\right)\\ LACR_{y}+l_{1}cos\left(\theta_{1}\right)+l_{2}cos\left(\theta_{1}+\theta_{2}\right)+l_{3}cos\left(\theta_{1}+\theta_{2}+\theta_{3}\right) \end{array}\right) \end{array};$$

(2) $$\begin{array}{c} \left(\begin{array}{c} RH_{x}\\ RH_{y} \end{array}\right)=\left(\begin{array}{c} RACR_{x}+l_{1}sin\left(\theta_{4}\right)+l_{2}sin\left(\theta_{4}+\theta_{5}\right)+l_{3}sin\left(\theta_{4}+\theta_{5}+\theta_{6}\right)\\ RACR_{y}-l_{1}cos\left(\theta_{4}\right)-l_{2}cos\left(\theta_{4}+\theta_{5}\right)-l_{3}cos\left(\theta_{4}+\theta_{5}+\theta_{6}\right) \end{array}\right) \end{array};$$

(3) $$\left(\begin{array}{c} LH_{y}\\ LH_{z} \end{array}\right)=\left(\begin{array}{c} LACR_{y}+l_{1}cos\left(\theta_{7}\right)+l_{2}cos\left(\theta_{7}+\theta_{8}\right)+l_{3}cos\left(\theta_{7}+\theta_{8}+\theta_{9}\right)\\ LACR_{z}+l_{1}sin\left(\theta_{7}\right)+l_{2}sin\left(\theta_{7}+\theta_{8}\right)+l_{3}sin\left(\theta_{7}+\theta_{8}+\theta_{9}\right) \end{array}\right);$$

(4) $$\left(\begin{array}{c} RH_{y}\\ RH_{z} \end{array}\right)=\left(\begin{array}{c} RACR_{y}-l_{1}cos\left(\theta_{10}\right)-l_{2}cos\left(\theta_{10}+\theta_{11}\right)-l_{3}cos\left(\theta_{10}+\theta_{11}+\theta_{12}\right)\\ RACR_{z}+l_{1}sin\left(\theta_{10}\right)+l_{2}sin\left(\theta_{10}+\theta_{11}\right)+l_{3}sin\left(\theta_{10}+\theta_{11}+\theta_{12}\right) \end{array}\right).$$

The relative orientation (*PV* = *LH* − *RH*) was then used as the hypothesized performance variable (*PV*) in the UCM analysis in both planes. This is clearly a redundant system because the number of degrees of freedom of the *PV* (*m* = 2) is much smaller than that of the elemental variable (*n* = 10). For the horizontal plane, the Jacobian matrix (*J*) which related differential changes in the elemental variables (*EV*), $dEV^{T}=\left(\begin{array}{cccccccccc} dLACR_{x} & dLACR_{y} & dRACR_{x} & dRACR_{y} & d\theta_{1} & d\theta_{2} & d\theta_{3} & d\theta_{4} & d\theta_{5} & d\theta_{6} \end{array}\right)$, to differential changes in the performance variable, (dPV=J⋅dEV), was as follows:

(5) $$J^{T}=(\begin{array}{cc} 1 & 0\\ 0 & 1\\ -1 & 0\\ 0 & -1\\ l_{1}cos\left(\theta_{1}\right)+l_{2}cos\left(\theta_{1:2}\right)+l_{3}cos\left(\theta_{1:3}\right) & -l_{1}sin\left(\theta_{1}\right)-l_{2}sin\left(\theta_{1:2}\right)-l_{3}sin\left(\theta_{1:3}\right)\\ l_{2}cos\left(\theta_{1:2}\right)+l_{3}cos\left(\theta_{1:3}\right) & -l_{2}sin\left(\theta_{1:2}\right)-l_{3}sin\left(\theta_{1:3}\right)\\ l_{3}cos\left(\theta_{1:3}\right) & -l_{3}sin\left(\theta_{1:3}\right)\\ -l_{1}cos\left(\theta_{4}\right)-l_{2}cos\left(\theta_{4:5}\right)-l_{3}cos\left(\theta_{4:6}\right) & -l_{1}sin\left(\theta_{4}\right)-l_{2}sin\left(\theta_{4:5}\right)-l_{3}sin\left(\theta_{4:6}\right)\\ -l_{2}cos\left(\theta_{4:5}\right)-l_{3}cos\left(\theta_{4:6}\right) & -l_{2}sin\left(\theta_{4:5}\right)-l_{3}sin\left(\theta_{4:6}\right)\\ -l_{3}cos\left(\theta_{4:6}\right) & -l_{3}sin\left(\theta_{4:6}\right) \end{array}).$$

Notation used to save space: $cos\left(\theta_{1:2}\right)=cos\left(\theta_{1}+\theta_{2}\right)$, $cos\left(\theta_{1:3}\right)=cos\left(\theta_{1}+\theta_{2}+\theta_{3}\right)$, etc.

Similarly, for the geometric model in the vertical plane, $dEV^{T}=\left(\begin{array}{cccccccccc} dLACR_{y} & dLACR_{z} & dRACR_{y} & dRACR_{z} & d\theta_{7} & d\theta_{8} & d\theta_{9} & d\theta_{10} & d\theta_{11} & d\theta_{12} \end{array}\right),$ and

(6) $$J^{T}=(\begin{array}{cc} 1 & 0\\ 0 & 1\\ -1 & 0\\ 0 & -1\\ -l_{1}sin\left(\theta_{7}\right)-l_{2}sin\left(\theta_{7:8}\right)-l_{3}sin\left(\theta_{7:9}\right) & l_{1}cos\left(\theta_{7}\right)+l_{2}cos\left(\theta_{7:8}\right)+l_{3}cos\left(\theta_{7:9}\right)\\ -l_{2}sin\left(\theta_{7:8}\right)-l_{3}sin\left(\theta_{7:9}\right) & l_{2}cos\left(\theta_{7:8}\right)+l_{3}cos\left(\theta_{7:9}\right)\\ -l_{3}sin\left(\theta_{7:9}\right) & l_{3}cos\left(\theta_{7:9}\right)\\ -l_{1}sin\left(\theta_{10}\right)-l_{2}sin\left(\theta_{10:11}\right)-l_{3}sin\left(\theta_{10:12}\right) & l_{1}cos\left(\theta_{10}\right)+l_{2}cos\left(\theta_{10:11}\right)+l_{3}cos\left(\theta_{10:12}\right)\\ -l_{2}sin\left(\theta_{10:11}\right)-l_{3}sin\left(\theta_{10:12}\right) & l_{2}cos\left(\theta_{10:11}\right)+l_{3}cos\left(\theta_{10:12}\right)\\ -l_{3}sin\left(\theta_{10:12}\right) & l_{3}cos\left(\theta_{10:12}\right) \end{array}).$$

To analyze the structure of the movement variability, we decomposed the total variability into two components, one component in the UCM (*V_UCM_*, variability that results in a net zero change of the performance variable, i.e., “goal-equivalent variability”) and the second component orthogonal to the UCM (*V_ORT_*, variability which results in a changed orientation of the arrow, i.e., “non-goal equivalent variability”) [13]. The equations below provide a dense notation in linear matrix algebra to compute the total variability and the components along the UCM and orthogonal to it [20] (***C*** is the covariance matrix of the elemental variables, and *null*(*J*) is an orthonormal basis of the Jacobian which can be computed with singular-value decomposition).

(7) $$V_{TOT}=\frac{trace\left(C\right)}{n\cdot N_{trials}};$$

(8) $$V_{UCM}=\frac{trace\left(null{\left(J\right)}^{T}\cdot C\cdot null\left(J\right)\right)}{\left(n-m\right)\cdot N_{trials}};$$

(9) $$V_{ORT}=\frac{trace\left({\left(J\cdot J^{T}\right)}^{-1}\cdot J\cdot C\cdot J^{T}\right)}{m\cdot N_{trials}}.$$

These equations were solved at every time point in the dataset (the Jacobian and covariance matrix are time-dependent according to the changing posture), resulting in a time series of *V_UCM_* and *V_ORT_.* This calculation was performed separately for the trials with different accuracy scores. The normalizations in the denominator by the dimensions of the space (*n* = 10: dimension of the elemental variable, and *m* = 2: dimension of the performance variable) and the number of trials in a particular condition were to be able to compare the values across participants and conditions.

For each plane of motion and accuracy score, we formulated two hypotheses about the control of the performance variable. The first hypothesis took the time dependence of the UCM into account, i.e., the covariance matrix was calculated around the time-dependent mean of the elemental variables, i.e., $C_{ii}\left(t\right)=1/N\cdot \sum_{Ntrials}{\left(EV{\left(t\right)}_{i}-{\bar{EV\left(t\right)}}_{i}\right)}^{2}$. This hypothesis assumes that the motor control system tries to attain different positions at different percentages of the aiming phase, for instance, a more closed position toward the point of release. The second hypothesis assumed a simplified version, namely a time-independent control, and we used the overall mean around which the covariance matrix was calculated, i.e., instead of ${\bar{EV\left(t\right)}}_{i}$, we used ${\bar{EV}}_{i}$ (mean across trials and throughout time). This hypothesis was motivated by visual inspection of the time series of the elemental variables in the aiming phase revealing no clear trends in the data.

For both hypotheses, planes of motion and accuracy conditions, we calculated *V_TOT_*, *V_UCM_*, and *V_ORT_*, and used these to define the index of motor abundance (IMA) [21]:

(10) $$\mathrm{IMA}=\frac{V_{UCM}-V_{ORT}}{V_{TOT}}.$$

This process up to the calculation of the index was performed for every subject individually and per accuracy score. A positive IMA (*V_UCM_* > *V_ORT_*) signifies that the elemental variables are controlled together in a specific way so as to stabilize the performance variable (kinematic synergy), while a negative IMA (*V_ORT_* > *V_UCM_*) indicates the reverse, i.e., destabilization of the performance variable. An IMA of zero would mean that there is no structure in the covariance of the elemental variables with respect to the chosen performance variable [21]. Similar to Reference [18], we took a criterion of a minimum of 10 trials per accuracy score to calculate a stable mean and covariance matrix around which the Jacobean could be linearized. However, the analysis of the accuracy (see Table 1) showed that the distribution was too skewed and unequal across subjects and did not meet this criterion of a minimum of 10 trials per score. We, therefore, concatenated all trials with scores of 6, 7, or 8 into one category (“low accuracy”) and all trials with scores of 9 or 10 into another category (“high accuracy”).

### 2.5. Statistical Analysis

To analyze the structure of the postural variability throughout the aiming phase, we performed two sets of tests with statistical parametric mapping (SPM) [22,23]. Firstly, we constructed mean IMA time series and confidence bands under the null hypothesis that the mean was zero ($H_{0}:\bar{\mathrm{IMA}}\left(t\right)=0$). When the lower bound of the confidence band excludes zero, this effectively means that the proposed performance variable was, in fact, controlled by systematic covariations in the elemental variables. This was performed for both hypotheses (time-independent and time-dependent control), in both planes (horizontal and vertical) and for both accuracy conditions (high and low). Secondly, we tested whether the postural control of trials with high accuracy had, in fact, relatively more variability within the UCM compared to trials of low accuracy. We constructed confidence bands for the paired difference in IMA between low- and high-accuracy trials under the null hypothesis that there was no difference ($H_{0}:{\bar{\mathrm{IMA}}}_{high}\left(t\right)={\bar{\mathrm{IMA}}}_{low}\left(t\right)$). This was performed for both planes of motion, as well as time-(in)dependent models. All SPM tests were conducted with the open-source spm1d-package for MATLAB [22] (© T. Pataky, spm1d.org). The level of type I error control was set nominally at 5% with Bonferroni corrections to preserve a family-wise 5% error rate within each set of tests.

## 3. Results

### 3.1. Shooting Accuracy

Table 1 presents the distribution of shooting accuracy per archer. The average accuracy over the 90 shots was situated between 8.22 (weakest performance, S6) and 8.94 (best performance, S4), and the distribution varied considerably. The same rank order of the archers was seen in the total score. Archers S1–4 had their mode on score 9 and archers S5–6 on score 8. Archer S4 was very stable and scored only 8, 9, or 10, while archers S2 and S3 also shot in the 7-zone, and archers S1 and S6 also in the 6-zone.

### 3.2. Uncontrolled Manifold Analysis

**Figure 3:**
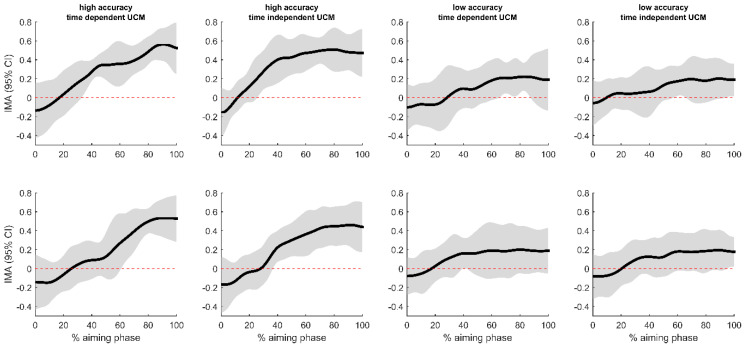
Time series of the mean index of motor abundance (IMA) and (1 − α)% confidence intervals (CIs) for the uncontrolled manifold (UCM) analyses. The upper panels give the UCM from the model in the horizontal plane (cfr. Figure 1a) and the lower panels for the UCM from the vertical plane (cfr. Figure 1b). (α = 0.00625).

Figure 3 shows the mean and 95% confidence intervals (CIs) for the IMA time series for the six archers, calculated for the geometric models in both planes and using the time-(in)dependent means for the trials with high and low accuracies. The overall patterns of the IMA trajectories are similar, starting at negative values and subsequently increasing. In all conditions and for all subjects, the IMA eventually reached positive values and this remained until the end of the aiming phase. In the trials with high accuracy, the 95% CIs lay completely above zero from some point in the aiming phase, indicating structural covariations between the elemental variables to stabilize the arrow’s orientation (*V_UCM_* >> *V_ORT_*). High-accuracy trials showed significant positive IMA values in the horizontal plane earlier in the aiming phase than for the vertical plane. The patterns appeared similar whether the Jacobean was linearized around the time-dependent or -independent means of the elemental variables. Additionally, for the trials with low accuracy, all subjects reached positive values, but these were smaller in magnitude, and the 95% CIs for the sample excluded zero only for brief parts of the aiming phase (*V_UCM_* ≈ *V_ORT_*). For the time-independent UCM, the 95% CIs barely excluded zero at the last two points before release.

**Figure 4:**
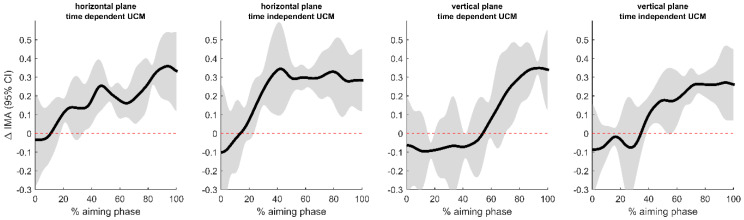
Paired sample (1 − α)% CIs for the difference in the IMA trajectories between trials of high and low accuracy. Positive values mean higher IMA for the high-accuracy trials. The CI bands effectively exclude zero during a large part of the aiming phase, indicating significantly higher values for the trials with higher accuracy. (α = 0.0125).

Figure 4 shows the results for the difference in IMA between high- and low-accuracy trials for both planes of motion and time-dependent and -independent means. The results corroborate the interpretation from Figure 3, specifically that the IMA is significantly higher for trials with high accuracy.

## 4. Discussion

### 4.1. Postural Control and Accuracy

This study aimed to provide deeper insight into the relationship between postural control during the aiming phase and shooting accuracy in recurve archery. Using the uncontrolled manifold concept, we tested several hypotheses about the structure of postural control of the elemental variables (lower limb and trunk posture and joint orientations of the upper limbs) in relation to the performance variable (orientation of the arrow with respect to the target). The dependent variable used in the analyses was the index of motor abundance (IMA) [21] which measures how the total trial-to-trial variability is related to stabilizing covariations of the elemental variables (*V_UCM_*) and (random) destabilizing variations (*V_ORT_*). The results showed consistently that, for trials with high accuracy, the IMA was significantly higher than zero during a large part of the aiming phase (Figure 3). This indicates the presence of kinematic synergy between the elemental variables that stabilizes the orientation of the arrow [12]. The low-accuracy trials also presented kinematic synergy, as could be expected for elite-level archers; however, this synergy was much weaker.

Synergies employed for arrows of higher accuracy (score 9 or 10) were significantly stronger than those for the lower-accuracy arrows (score 6, 7, or 8) as seen in the SPM confidence intervals of the index of motor abundance (Figure 4). However, we have to keep in mind here the limitation of the archers having different accuracy distributions; thus, the present result is only a first approximation to the relationship between accuracy and the structure of postural control. All archers contributed trials with 9- and 10-scores to the high-accuracy set, but, for the low-accuracy set, not every archer contributed trials with 6- and 7-scores (archer S4, in fact, had only 8-scores). Nevertheless, the results illustrate a potential relationship that has to be examined further in large-scale studies. Postural control analyzed with UCM hypotheses has stronger internal logic than previous studies on the relationship between postural control and accuracy [8,9,10,11] because it effectively takes all relevant degrees of freedom into account simultaneously with respect to a performance variable. The present results are difficult to compare with previous studies because different analysis techniques were used. Previous studies observed both positive and negative associations between shooting accuracy and movement characteristics. However, when these characteristics (sway amplitudes, joint excursions, velocities, within- and between-trial variabilities, etc.) are examined in isolation from each other and in isolation from a performance variable, this does not necessarily mean that they are directly related to performance.

### 4.2. On the Nature of Stabilizing Kinematic Synergies in Archery

An exact description of synergy that stabilizes the arrow’s orientation is not possible, and it is even likely that the different archers employed different synergies depending on their intrinsic biomechanical properties, level of expertise, intentions, and possible other causes [13,18]. For instance, a subject with intrinsic unstable posture of the lower limbs and trunk might have learned to employ a strategy where the upper limb joints effectively counterbalance the perturbations of the trunk movements. Vice versa, for subjects with lower upper-body strength, the external forces on the bow and string hands produce strong perturbations on the arrow’s orientation which may be cancelled by appropriate covariation of trunk movements. While these disturbances and (unconscious) stabilizing covariations are (likely) not detectable to the naked eye, the present UCM analysis reveals that they exist and that there is a link to the shooting accuracy. The observation that all IMA time series started at negative values and eventually reached positive values for all participants and conditions may indicate that the motor control system cannot implement these synergies instantly at the point of full draw (start of the aiming phase), but that it guides the system toward the UCM. Once the system has found a stable position, the variance orthogonal to the UCM is decreased further, and as a result, the system decides to release the arrow. Releasing the arrow at a point when *V_UCM_* >> *V_ORT_* is efficient since the act of releasing the arrow will undoubtedly increase *V_ORT_* because the drop in external force on the draw hand may slightly affect the position of the bow hand before the arrow has completely left the bow.

While we performed no formal statistical test for the difference in IMA between the models in the vertical vs. horizontal plane, their results were qualitatively similar. The synergy stabilizing the arrow’s direction in the *xy*-plane was similar in strength to that in the *yz*-plane. The high-accuracy trials had 0.4 < IMA < 0.6 for both planes of motion at the time of release, and the low-accuracy trials had 0.1 < IMA < 0.3 for both planes of motion. Future studies may examine more explicitly whether postural control in either direction is favored over another or not and how this affects shooting accuracy, but this was not the aim of the present study. The same comment applies for the comparison of time-dependent vs. -independent postural control, which gave qualitatively similar results. A parsimonious interpretation of this observation hints toward the control being independent of the time during the aiming phase, but would require further research to test this explicitly. However, a sample-level statistical test like that performed in this study could not be performed to test this, since the time dependency of the UCM is not necessarily the same in different subjects.

An important question arises as to how these synergies are activated by the motor control system and how they evolve during motor learning. Particularly for a static task like archery shooting, the question asked by Latash is very relevant: “So why would the controller increase the variance of elemental variables, and at the same time, organize a synergy stabilizing their combined effect? Would it not be easier to keep the elemental variance unchanged and not worry about synergies?” [24]. Why does the motor control system not minimize every degree of freedom’s movement? He proposed two possible answers, namely the ability to perform secondary tasks with the same elemental variables without detrimental effects on the performance variable of the primary task and to be able to handle unexpected perturbations [24]. The first answer is unlikely to be relevant in archery and other precision shooting sports as secondary tasks are absent. While the rules of archery purposefully try minimizing all external perturbations (noise, light, external forces), the second answer is better suited because small internal perturbations (muscle tremors, competition stress, thoughts, etc.) are unavoidable and will affect the orientation of the arrow.

These kinematic synergies should be implemented by certain neural systems, and studies on brain dynamics in archery shooting may provide clues in this direction. Seo et al. [25] used a judgement-of-line-orientation task (similar to aiming) with experienced and novice archers while measuring brain activation and deactivation patterns with functional magnetic resonance imaging (fMRI). Expert archers exhibited higher task-related cortical activations related to visuospatial attention and working memory, as well as simultaneous stronger task-related deactivations in other cortical areas, compared to the novices. In relation to accuracy, they found a positive correlation (r = 0.32) between success rate and change of middle-frontal gyrus activity and a negative correlation (r = −0.31) between success rate and change of paracentral cortex/precuneus activity. Similarly, Kim et al. observed different fMRI activity in world-class and novice archers during the pre-performance routine period of a virtual aiming task [26]. In yet another fMRI study, experienced archers not only exhibited more localized cortical activity than novices, but also higher activity in the cerebellar dentate, which the authors interpreted as evidence for the cerebellum’s involvement in automating simultaneous movements and integration of sensorimotor memory [27], which fits within the explanation of the results using synergies. How exactly these different brain activity patterns are acquired during learning and their relationships with the learning of kinematic synergies are unknown.

On an applied level, teaching archers to employ stabilizing synergies can be performed with challenging tasks. Wu and Latash recommend using non-repetitive tasks to prevent stereotypical solutions and manipulating stability conditions to encourage larger amounts of “good variability” (*V_UCM_*) [28]. In a multi-finger force production task, this strategy effectively leads to larger amounts of *V_UCM_* and smaller amounts of *V_ORT_* after practice. For archery training, this could mean that, in addition to training regimes under normal conditions (no distractions, standard execution), exercises could be implemented that require the archer to shoot from various (unstable) positions and joint configurations (e.g., differential learning [29]). This learning strategy is yet to be tested in archery training and should be evaluated prior to implementation in elite-level practice.

### 4.3. Study Limitations and Further Research

A first limitation is a small sample size which limits generalizability and the lack of sufficient trials with the lowest and highest accuracies in most archers (score 6, 7, and 10). This forced us to concatenate the trials into only two categories, which decreased the information that the data provided. Nevertheless, the two categories still distinguished the postural control between trials of high and low accuracy, but it should be kept in mind that the different archers contributed different distributions of shots to each category. Two possibilities to circumvent this issue in future research are to repeat this experiment on multiple days until sufficient data for each category is collected or to use a more heterogeneous population of archers and perform a linear regression analysis between the mean/total score and the IMA trajectory. This was not feasible in the present sample because the range of mean/total score was too restricted to be meaningful for regression analysis.

A second limitation is the time normalization issue. Because the motion capturing was not synchronized with the clicker device on the bow, the true point of release had to be estimated indirectly from the kinematic data. Although the method appeared consistent, there may still be random errors in this method which caused variability in the timing of the start and end phase, and correspondingly, in the joint-angle time series. Scholz et al. [18] say this variability may contribute to either the *V_UCM_* or *V_ORT_* with unknown probabilities, and that there is no particular reason to expect the error to contribute to either or both. In the present static task, we also believe that this would not cause errors in the analyses, especially for the time-independent UCM.

It was also unknown whether the selected performance variable of relative hand orientations was an unbiased estimator of the true arrow orientation because the arrow head could not be tracked with the 3D motion capturing. In future studies, it would be interesting to additionally include analysis of upper limb muscle synergies (electromyography) in addition to the kinematic synergies examined in the present study. Such a multi-level view on motor control can provide more information on the mechanisms responsible for the presently observed results.

## Figures and Tables

**Table 1:** Distribution of shooting accuracy for all six archers.

	Score 6	Score 7	Score 8	Score 9	Score 10	Mean ± SD	Total Score
S1	2	6	6	66	10	8.84 ± 0.79	796
S2	0	5	17	65	3	8.73 ± 0.61	786
S3	0	3	9	72	6	8.90 ± 0.54	801
S4	0	0	10	75	5	8.94 ± 0.41	805
S5	1	7	39	30	13	8.52 ± 0.88	767
S6	1	14	45	24	6	8.22 ± 0.83	740

## References

[1] Ertan H., Soylu A.R., Korkusuz F. (2005). Quantification the relationship between FITA scores and EMG skill indexes in archery. J. Electromyogr. Kinesiol..

[2] Ertan H. (2009). Muscular activation patterns of the bow arm in recurve archery. J. Sci. Med. Sport.

[3] Soylu A.R., Ertan H., Korkusuz F. (2006). Archery performance level and repeatability of event-related EMG. Hum. Mov. Sci..

[4] Ertan H., Kentel B., Tümer S.T., Korkusuz F. (2003). Activation patterns in forearm muscles during archery shooting. Hum. Mov. Sci..

[5] Keast D., Elliott B. (1990). Fine body movements and the cardiac cycle in archery. J. Sports Sci..

[6] Callaway A.J., Wiedlack J., Heller M. (2016). Identification of temporal factors related to shot performance for indoor recurve archery. J. Sport Sci..

[7] Nabavinik M., Abaszadeh A., Mehranmanesh M., Rosenbaum D.A. (2018). Especial skills in experienced archers. J. Mot. Behav..

[8] Mohamed M.N., Azhar A.H. (2012). Postural sway and shooting accuracy of skilled recurve archers. Mov. Health Exerc..

[9] Spratford W., Campbell R. (2017). Postural stability, clicker reaction time and bow draw force predict performance in elite recurve archery. Eur. J. Sport Sci..

[10] Tinazci C. (2011). Shooting dynamics in archery: A multidimensional analysis from drawing to releasing in male archers. Procedia Eng..

[11] Stuart J., Atha J. (1990). Postural consistency in skilled archers. J. Sports Sci..

[12] Scholz J.P., Schöner G. (1999). The uncontrolled manifold concept: Identifying control variables for a functional task. Exp. Brain Res..

[13] Latash M.L. (2010). Motor synergies and the equilibrium-point hypothesis. Mot. Control.

[14] Feldman A.G., Levin M.F., Sternad D. (2016). The Equilibrium-Point Hypothesis—Past, Present and Future. Progress in Motor Control.

[15] Feldman A.G. (2011). Space and time in the context of equilibrium-point theory. Wiley Interdiscip. Rev. Cogn. Sci..

[16] Latash M.L. (2018). Abundant Degrees of Freedom Are Not a Problem. Kinesiol. Rev..

[17] Latash M.L., Scholz J.P., Schöner G. (2007). Towards a new theory of motor synergies. Mot. Control.

[18] Scholz J.P., Schoner G., Latash M.L. (2000). Identifying the control structure of multijoint coordination during pistol shooting. Exp. Brain Res..

[19] Balasubramaniam R., Riley M., Turvey M.T. (2000). Specificity of postural sway to the demands of a precision task. Gait Posture.

[20] Yen J.T., Chang Y.-H. (2010). Rate-dependent control strategies stabilize limb forces during human locomotion. J. R. Soc. Interface.

[21] Tseng Y., Scholz J.P. (2005). The effect of workspace on the use of motor abundance. Mot. Control.

[22] Pataky T.C. (2010). Generalized n-dimensional biomechanical field analysis using statistical parametric mapping. J. Biomech..

[23] Pataky T.C. (2012). One-dimensional statistical parametric mapping in Python. Comput. Methods Biomech. Biomed. Eng..

[24] Latash M.L. (2010). Stages in learning motor synergies: A view based on the equilibrium-point hypothesis. Hum. Mov. Sci..

[25] Seo J., Kim Y.-T., Song H.-J., Lee H.J., Lee J., Jung T.-D., Lee G., Kwon E., Kim J.G., Chang Y. (2012). Stronger activation and deactivation in archery experts for differential cognitive strategy in visuospatial working memory processing. Behav. Brain Res..

[26] Kim J., Lee H.M., Kim W.J., Park H.J., Kim S.W., Moon D.H., Woo M., Tennant L.K. (2008). Neural correlates of pre-performance routines in expert and novice archers. Neurosci. Lett..

[27] Kim W., Chang Y., Kim J., Ryu K., Lee E., Woo M., Janelle C.M. (2014). An fMRI study of differences in brain activity among elite, expert and novice archers at the moment of optimal timing. Cogn. Behav. Neurol..

[28] Wu Y.-H., Latash M.L. (2014). The effects of practice on coordination. Exerc. Sport Sci. Rev..

[29] Schöllhorn W.I., Beckmann H., Davids K. (2010). Exploiting system fluctuations. Differential training in physical prevention and rehabilitation programs for health and exercise. Med. Kaunas.

